# Enhanced T-lymphocyte infiltration in a desmoid tumor of the thoracic wall in a young woman treated with intratumoral injections of the oncolytic peptide LTX-315: a case report

**DOI:** 10.1186/s13256-019-2088-6

**Published:** 2019-06-10

**Authors:** Nina Louise Jebsen, Torunn Oveland Apelseth, Hans Kristian Haugland, Øystein Rekdal, Hamina Patel, Bjørn Tore Gjertsen, Dag Eirik Jøssang

**Affiliations:** 1 Centre for Cancer Biomarkers (CCBIO), Department of Clinical Science, University of Bergen, Bergen, Norway; 2 Department of Oncology, Haukeland University Hospital, Bergen, Norway; 3 Department of Medical Biochemistry and Pharmacology, Haukeland University Hospital, Bergen, Norway; 4 Department of Immunology and Transfusion Medicine, Haukeland University Hospital, Bergen, Norway; 5 Department of Pathology, Haukeland University Hospital, Bergen, Norway; 6 Lytix Biopharma and Molecular inflammation research group (MIRG), University of Tromsø, Tromsø, Norway; 7 Department of Internal Medicine, Hematology Section, Haukeland University Hospital, Bergen, Norway; 8 Department of Radiology, Haukeland University Hospital, Bergen, Norway

**Keywords:** Desmoid tumors, Intratumoral oncolytic peptide, T-cell infiltration, Immune gene expression profiles

## Abstract

**Background:**

Desmoid tumors are intermediary malignant, fibrous lesions occurring in various soft tissues. Surgical treatment is relentlessly challenging because of the propensity for local aggressive behavior and high risk of recurrence. Consequently, a wide range of oncological drugs and radiation therapy are being used; however, outcomes are unpredictable. We investigated whether local treatment with an oncolytic peptide could be beneficial in a patient with an unresectable desmoid tumor.

**Case presentation:**

In a young 29-year-old Caucasian woman who was diagnosed with a retromammary desmoid tumor infiltrating deeply into the anterior thoracic wall, surgery was considered excessively mutilating, and observation was recommended. The lesion progressed, however, and caused debilitating pain, despite nonsteroidal anti-inflammatory medication. Subcutaneous injections of human interferon-α (Multiferon®) resulted in reduced growth kinetics but had to be terminated because of development of symptomatic pneumonitis. Frequently used oncological treatment was withheld because of the toxicity profile, and the patient was instead included in a phase I study investigating transdermal intratumoral injection of LTX-315, an oncolytic peptide that induces anticancer immune responses (ClinicalTrials.gov, NCT01986426). A marked increase of CD8^+^ tumor-infiltrating T cells in the lesion was complemented by upregulation of immune gene signature (including effector T-cell, T-helper type 1 cell, chemokine, and cytokine genes). These changes were followed by gradual symptom relief and long-term disease stabilization, indicating clinical benefit. LTX-315 was well tolerated until termination in week 16 after a serious allergic reaction.

**Conclusions:**

Our patient was treated with repeated intratumoral injections of LTX-315, resulting in tumor regression accompanied by upregulation of immune genes and T-cell infiltration. Local application of immunotherapy, minimizing systemic side effects, represents a novel treatment modality in desmoid tumors that should be tested in further clinical trials.

## Background

Desmoid tumors (DTs) or aggressive fibromatoses are rare, with a yearly incidence of 2–5 per 1 million population. Not apt to metastasize, they represent locally infiltrating soft tissue tumors that may occur anywhere in the body [1]. A seemingly multifactorial pathogenesis, including physical trauma, endocrine factors, and genetic aspects, induces a proliferation of fibroblasts, resulting in extremely hard, fibrotic lesions [2], approximately 90% of which comprise a somatic mutation of β-catenin (*CTNNB1*) [3]. The peak incidence is in the second to fourth decades of life, and a preponderance of the patients are female; otherwise, the sex incidence is equal in pediatric and older patients. The clinical development varies greatly from incidental findings of indolent course to progressive and mutilating disease imposing pain, major functional deficits, and even death in the case of intra-abdominal location. Symptomatic lesions call for action; however, treatment is challenging and often requires a trial-and-error approach [4–6]. Owing to the confined nature of the disease, it seems rational to favor local treatment. Surgery often necessitates extensive resections and is associated with a high risk of local recurrence; hence, surgical excision is no longer considered a mainstay strategy [4, 5]. Radiotherapy entails a risk of severe late morbidity and secondary cancer. Recommended medications range from nonsteroidal anti-inflammatory drugs (NSAIDs) and endocrine treatment to kinase inhibitors and toxic chemotherapy [7–11]. Immune therapy with recombinant interferon-α (IFN-α) or a human leukocyte derived mixture of naturally occurring IFN-α subtypes (Multiferon®) has been described as effective in selected cases [12, 13].

Relative contraindications to frequently used treatment options in a case of a young woman with a retromammary DT motivated the investigation of intratumoral injection of an oncolytic peptide within an ongoing phase I study in solid tumors. Long-term clinical benefit was achieved.

## Case presentation

A young Caucasian woman (aged 29 years) was diagnosed in January 2013 with a DT (5 cm in longest diameter) infiltrating deeply into the left thoracic wall and involving multiple layers of soft tissue. Her only symptom was a palpable lesion; she had no pain. Surgery aiming for a wide safety margin would have required a massive resection and was considered contraindicated. During surveillance, the lesion gradually enlarged to approximately 8 cm, accompanied by increasing aching and tenderness. In addition, the patient experienced attacks of intense neuropathic pain causing frequent nocturnal awakenings, as well as restricted function of her left arm. Progressing tumor and debilitating symptoms compelled medical treatment. NSAIDs partially reduced pain but not tumor growth. Endocrine therapy with tamoxifen was discarded because the patient wanted a second child, and teratogenic effects have been reported and may persist as a risk many months after cessation of tamoxifen [14]. Instead, on the basis of Scandinavian experience, the patient received human IFN-α (Multiferon®) at a dose of 3 million IU daily administered subcutaneously in the period from May through August 2014 [12, 13]. Radiological evaluation demonstrated reduced growth kinetics and disease stabilization. However, the patient developed pneumonitis, and Multiferon® had to be stopped prematurely. Her pain did not diminish, but it was partially alleviated by ibumetin and later celecoxib in combination with paracetamol. Despite receiving pain relievers, she woke up approximately ten times every night and developed symptoms of sleep deprivation. Her young age and the retromammary localization of the tumor informed her reluctance to undergo radiotherapy, owing to a long-term risk of secondary breast cancer. Tyrosine kinase inhibitors have demonstrated promising progression-free survival, but only rarely has a partial regression pertinent to mitigating symptoms been achieved. Moreover, stable disease was already achieved with Multiferon®. Because the patient responded to immunotherapy with IFNs, we postulated that her tumor also might respond to intratumoral immunotherapy. She was therefore recruited to undergo local injection of an oncolytic peptide (LTX-315; Lytix Biopharma, Oslo, Norway) in an ongoing phase I study of solid tumors (ClinicalTrials.gov, NCT01986426) [15, 16].

### Investigations

Initially, the patient was examined at a breast diagnostic center. Core biopsy revealed a fibrous lesion of spindle-shaped cells in a fascicular pattern with uniform nuclei. No epithelial components were evident. IHC demonstrated strong positivity for vimentin in addition to positivity for β-catenin and actin 1A4, slight positivity for desmin, and focal positivity for actin HHF35. Weak positivity for estrogen was spotted in single endothelial cells, although not in spindle-shaped cells. Ki67 expression was < 5%. Altogether, the findings supported a diagnosis of extra-abdominal DT. Additional molecular diagnostics detected a mutation in exon 3 of *CTNNB1*, codon 45 (S45F).

Diagnostic magnetic resonance imaging (MRI) and baseline MRI prior to LTX-315 demonstrated an irregularly shaped soft tissue mass with a moderate contrast uptake situated close to the axillary process and cranial component of the left breast, infiltrating dorsolateral parts of the pectoralis minor and major muscles, as well as anterior parts of the serratus, latissimus dorsi, and intercostal muscles. In the superficial direction, the lesion infiltrated the subcutaneous fatty tissue.

**Fig. 1:**
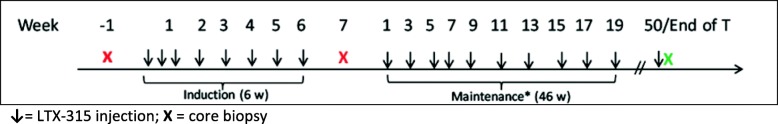
This diagram has been provided to the author by Lytix Biopharma and approved for use in this article by medical chief officer as well as co-founder of Lytix Biopharma

Positron emission tomography/computed tomography demonstrated only a minor fluorodeoxyglucose uptake (maximum standardized uptake value, 3.2) in the lesion and was considered unsuitable for evaluation. Baseline Ultrasound (US) was used to guide LTX-315 injections and for continuous evaluation during the study, in addition to MRI. Baseline Ultrasound (US) revealed a nonsymmetric and diffusely demarcated lesion. In line with the phase I protocol, research core biopsies were undertaken at baseline (week − 1), after induction (week 7), and at the end of treatment (week 18) (Fig. 1).

Tumor tissue was evaluated by H&E (Haemotoxylin and Eosin) staining as well as staining of CD3^+^ and CD8^+^ T cells in the pretreatment biopsy, following induction, and at the end of treatment. Next-generation sequencing of the β-T-cell receptor genes was applied in order to detect clonal expansion of tumor-associated peripheral blood mononuclear T cells in blood, including clones that also were present in the tumor. Identification of upregulated key immune genes involved in tumor regression was carried out by hierarchical clustering of Immunosign 21 immune gene signature (HalioDx, Richmond, VA, USA) by means of a predefined set of genes, including effector T-cell, T-helper type 1 (Th1) cell, chemokine, and cytokine genes.

### Differential diagnosis

Upon referral to the hospital, the patient was informed that a primary breast cancer was suspected. However, in contrast to most breast carcinomas, the lesion was not detected by mammography. Other clinical differential diagnoses for DTs are a variety of fibromas, nodular fasciitis, or soft tissue sarcoma. Histopathologically, the most frequent misclassifications are Gardner fibroma, scar tissue, superficial fibromatosis, nodular fasciitis, myofibroma, collagenous fibroma, and low-grade fibromyxoid sarcoma [17].

### Treatment

**Fig. 2:**
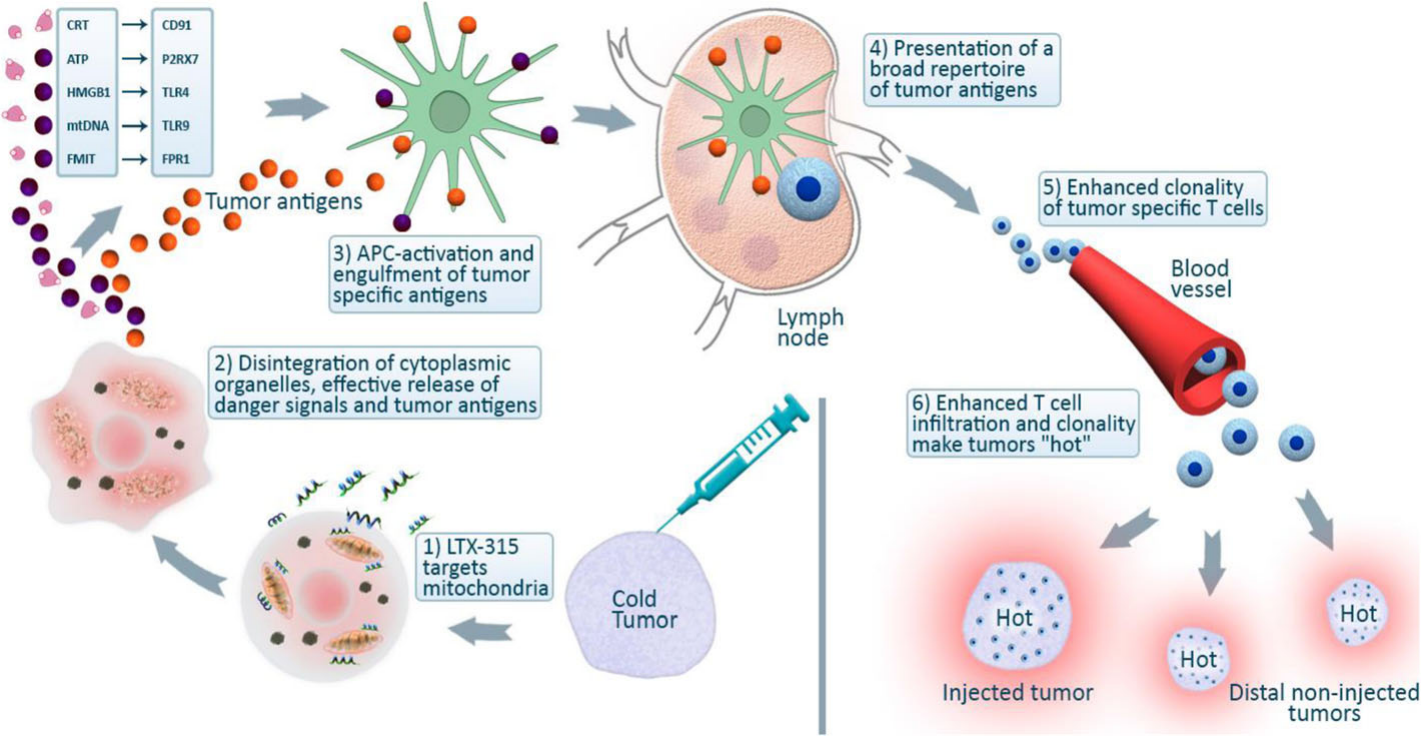
LTX-315 mode of action

LTX-315 is an oncolytic peptide derived from lactoferrin, a membrane host defense peptide. When injected into malignant tumors, LTX-315 targets mitochondria and prompts direct cell death by disintegrating cytoplasmic organelles [18]. Release of tumor antigens, in turn, induces specific systemic anti-tumor responses by enhanced T-cell clonality and an enhanced number of tumor-infiltrating T cells, transforming non-T-cell inflamed cold tumors into T-cell-inflamed hot targets (Fig. 2) [19]. This mechanism of action has been demonstrated in the current phase I study, from which clinical trial data are to be published.

LTX-315 (5 mg; 20 mg/ml) was injected under US guidance into the tumor tissue and deposed immediately inside the pseudocapsule in a clockwise manner for the following intervals: days 1 and 3 during the first week, once weekly thereafter for a total of 6 weeks, followed by a maintenance phase with one injection every second week until maintenance week 9 (Fig. 1). Local pain during and shortly after the injection varied from mild to severe; however, no pain medication was necessary, owing to only short-term discomfort. Allergy-like reactions such as hot flushes and mild edema of the face and arms were encountered after the first two injections but subsided until the 11th dose, after which the patient reported hot flushes and edema of her upper body, itching, nausea, hypertension, and tachycardia (all grade 1 adverse events according to National Cancer Institute Common Terminology Criteria for Adverse Events), in addition to grade 2 headache, dizziness, and swelling of the throat. The subsequent dose was therefore administered with premedication (antihistamines and dexamethasone) 12 hours and 1 hour prior to study drug application. Despite allergy prophylaxis, following the 12th dose, a grade 3 anaphylactic reaction occurred immediately after the injection, presenting with symptoms similar to those described above except more pronounced, including edema of the throat classified as grade 3. The patient’s respiratory distress was quickly relieved with subcutaneous administration of adrenaline (0.3 mg). All symptoms resolved nearly entirely within 30 minutes, and she was discharged from the Clinical Trial Unit the same afternoon. Apart from moderate tiredness for the next couple of days, she completely recovered. Evidently, this anaphylactic episode led to termination of the treatment.

### Outcome and follow-up

Following the anaphylactic reaction, supplementary tests were performed to investigate the underlying immunological mechanisms. The result of a skin prick test was negative, whereas the result of a subsequent intradermal skin test was considered positive. A transient rise in eosinophils shortly after the reactions was measured. Results of a basophil activation test and histamine release test were both interpreted as positive. Antibody investigation (manual enzyme-linked immunosorbent assay) revealed specific immunoglobulin G (IgG) antibodies against LTX-315, although no specific IgE antibodies were found. Specific IgG antibodies may form immune complexes with LTX-315, thereby activating complement. Complement activation factors such as C3a and C5a may directly activate basophils and mast cells, possibly explaining the test results.

**Fig. 3:**
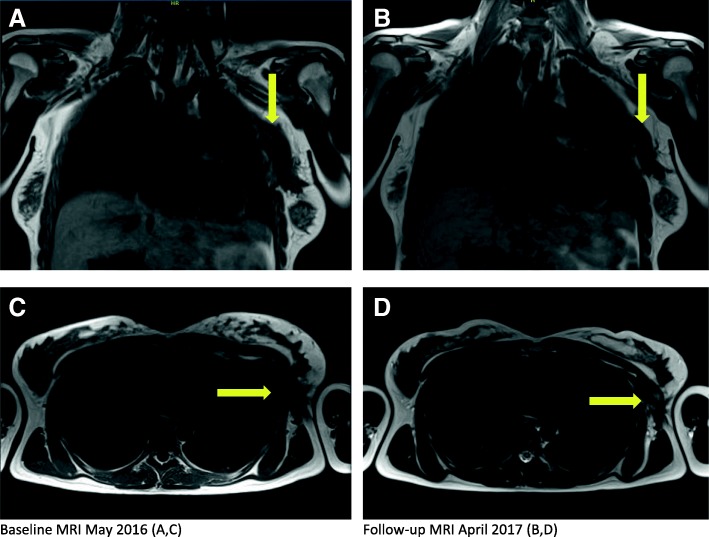
Radiological evaluation. Magnetic resonance imaging (t1 weighted) demonstrating frontal (**a**, **b**) (yellow arrows indicating cranial tumour border) and axial (**c**, **d**) (yellow arrows indicating medial tumour border) sections of pretreatment (**a**, **c**) and follow-up (**b**, **d**) examination 19 months after completion of intratumoural LTX-315 injections

Radiologically, the pretreatment tumor size in May 2015 was 8.6 × 7.9 × 2.9 cm based on MRI, corresponding to 3.6 cm in thickness, 8.1 cm craniocaudally, and 9.1 cm anteroposteriorly on a baseline US scan. By the time of the last injection, the lesion had decreased to 7.0 × 7.0 × 2.5 cm based on US. Similarly, the tumor had slightly decreased in size to 7.0 × 6.0 × 2.0 cm based on MRI, becoming clinically less prominent 4 months after the start of LTX-315 (Fig. 3). A reduction of the greatest tumor diameter by 19% qualified for stable disease according to RECIST 1.1 criteria (Response Evaluation Criteria in Solid Tumours). No morphologic changes were apparent on US scans during the study period.

**Fig. 4:**
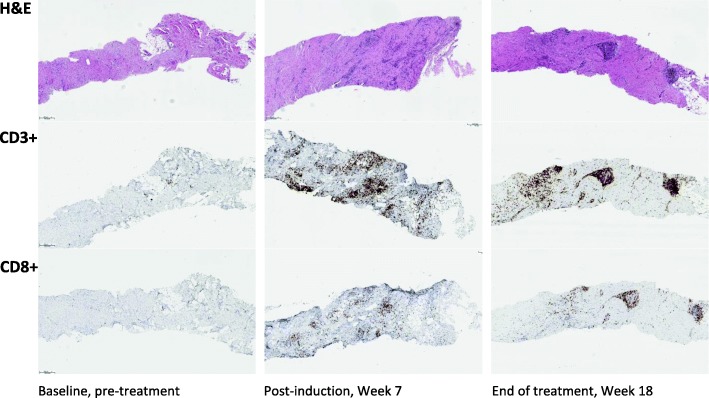
Pre- and post-treatment biopsies. Haemotoxylin and Eosin staining and staining of CD3^+^ and CD8^+^ T cells in pretreatment biopsy (left column), postinduction week 7 (middle column), and at the end of treatment 18 weeks later (right column)

**Fig. 5:**
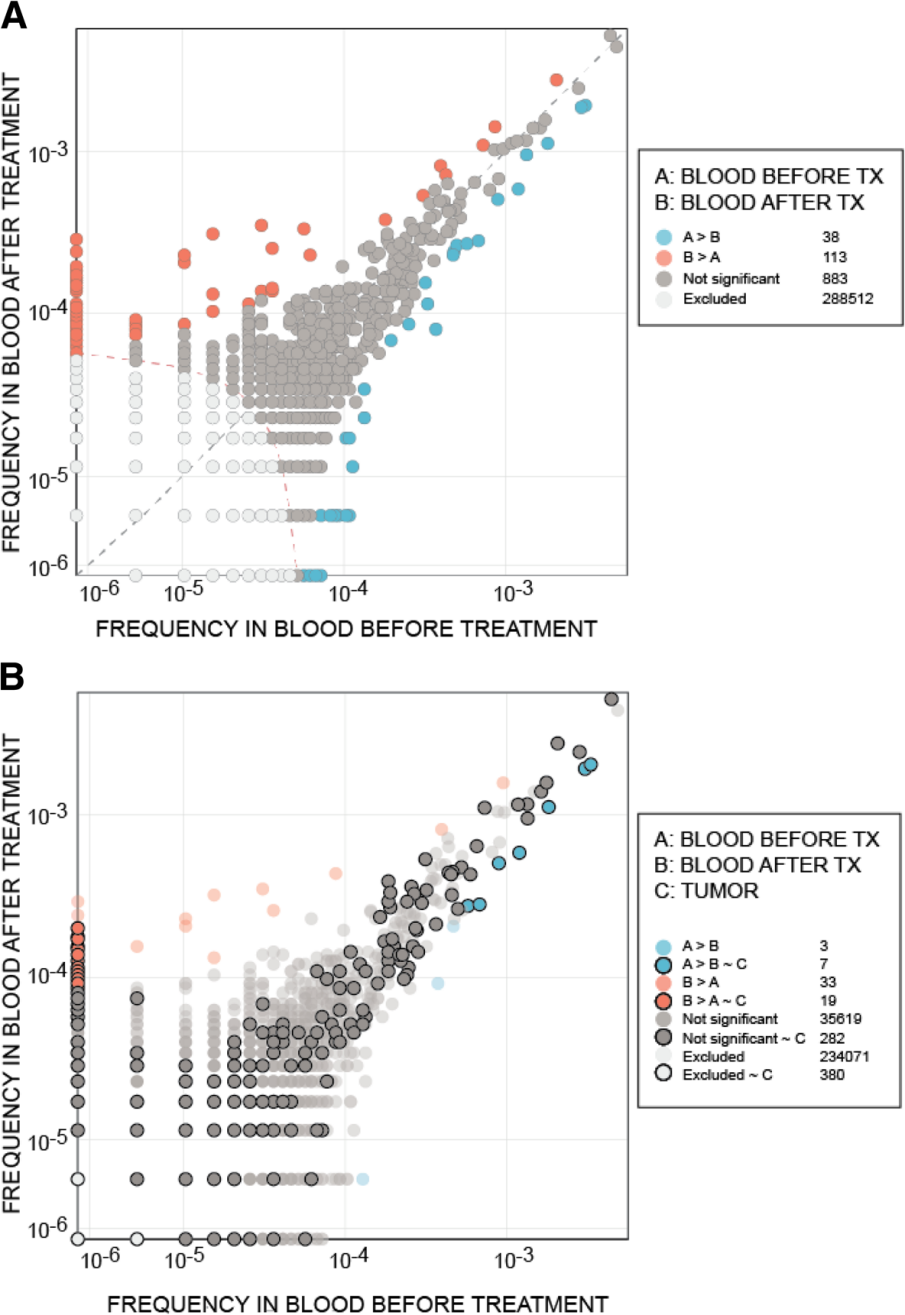
T-cell analyses. **a** Graph showing the abundance of unique T-cell clones in blood (peripheral blood mononuclear cells) before treatment compared with 7 weeks after initiation of LTX-315 treatment. T-cell clones that were not significantly changed in frequency are depicted in *gray*, whereas those that were significantly expanding or contracting are depicted in *red* or *blue*, respectively. *Dashed diagonal gray line* defines frequency equality, and *dashed red line* defines threshold for statistical comparison. **b** Trivariate analysis of T-cell clones in blood (similar analysis to that in **a**) compared with the T-cell clones in the tumor after LTX-315 treatment. T-cell clones colored without transparency and with a black peripheral line were also present in the tumor tissue

**Fig. 6:**
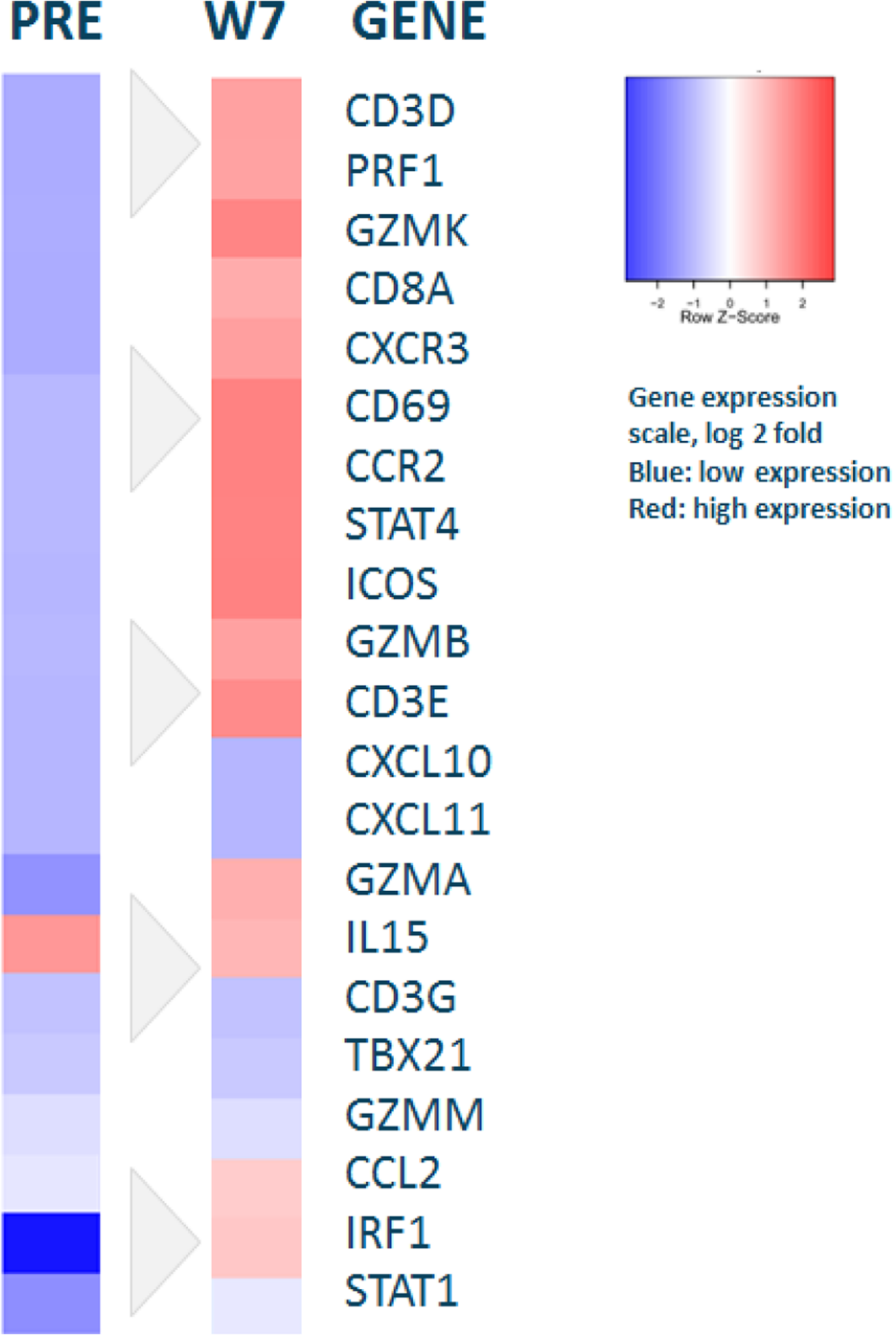
Gene expression profiles. LTX-315 induced upregulation of key genes involved in tumor regression and transformed the gene expression from cold (*blue*) to hot (*red*). Hierarchical clustering of Immunosign 21 immune gene signature (HalioDx) profiling a predefined set of effector T-cell, T-helper type 1 cell, chemokine, and cytokine genes

Histopathologically, abundant infiltration of CD3^+^ and CD8^+^ T cells was demonstrated in the biopsies obtained at week 7 and at the end of treatment (18 weeks from start of LTX-315) compared with the pretreatment biopsy done in May 2015 (Fig. 4). The number of CD3^+^ T cells in two different biopsies increased by a factor of 16.7 from averages of 76.7 and 43.0 per mm^2^ of tumor tissue at baseline to 817.1 and 1181.9 per mm^2^ of tumor tissue at 7 weeks. Similarly, the subpopulation of CD8^+^ T cells in the two biopsies at baseline and postinduction (week 7) increased approximately sevenfold from 74.2 and 32.1 to 329.4 and 422.8 cells per mm^2^ of tumor tissue. Altogether, 52 T-cell clones among peripheral blood mononuclear cells (PBMC) expanded significantly following drug exposure, 19 of which were present in the tumor tissue in the post-treatment biopsy (Fig. 5). By profiling a predefined set of effector T cell, Th1 cell, chemokine, and cytokine genes, a change from cold to hot gene expression confirmed upregulation of immune genes typically involved in tumor regression (Fig. 6).

Clinically, the patient’s complaints of short pulses of stinging pain of neuropathic character changed to more constant pain of lower intensity during the course of LTX-315 therapy. Except for the treatment days, the patient was able to attend work during the study period. The aching pain gradually decreased in amplitude over time, and by March 2016, she reported that she had stopped taking pain medication, hardly ever woke up during the night, and was able to resume playing handball. Concurrent MRI revealed that the tumor was shrinking slowly (6.5 × 6.1 × 1.9 cm), representing a tumor diameter reduction of 24% from baseline. The lesion has been stable based on subsequent follow-up MRI scans more than 2.5 years after the completion of study treatment.

## Discussion and conclusions

DTs are classified as intermediate malignant lesions because of their infiltrative behavior [1]. Surveillance is recommended in asymptomatic cases because surgery entails a high risk of local recurrence correlating with the quality of the surgical margin [20]. Treatment is challenging in cases of rapid progression, and the severity of the disease directs the treatment decision process [4–6]. When prompt response is crucial because of compromised function of vital organs, chemotherapy in oncological doses is typically the first choice [7]. Less dramatic development may motivate low-dose chemotherapy, IFN-α, or, more recently, tyrosine kinase inhibitors [8, 9]. Tamoxifen and NSAIDs (typically sulindac) are attempted in more indolent cases [10, 11].

Histopathological examination of DTs reveals uniform, spindle-shaped, small-nucleated cells within a collagen-rich stroma and variable prominent vessels or occasionally perivascular edema. Cellular atypia is absent [1]. Activating mutations of the *CTNNB1* gene are detected in the majority of sporadic DTs counteracting the normal inhibitory effects of adenomatous polyposis coli (APC) protein on the β-catenin complex, a downstream effector of which the intricate function involves pleiotropy, interaction with transcription factors, cell growth, and homeostatic processes [3]. β-Catenin plays a key role in the Wnt-signaling cascade responsible for embryonal formation of tissue and organ development, as well as in cell regulation and regeneration in adults.

DTs most frequently arise in the abdominal wall in young/middle-aged women, in whom there is an association with pregnancy. Other locations are deep-seated, poorly circumscribed fibromatosis of the trunk wall, girdles, and head and neck area. Individuals with familial adenomatous polyposis (FAP) harbor an increased susceptibility to develop mesenteric/intra-abdominal DTs because germline inactivation of the APC tumor suppressor gene found in patients with FAP interrupts APC/β-catenin binding, resulting in inhibition of the Wnt pathway [21].

In young females, endocrine treatment with tamoxifen is considered first choice by many [22]. When we informed our patient about potential teratogenic side effects, she rejected this option. Anti-inflammatory drugs are frequently administered, but with limited success in most patients, as in our patient. Chemotherapy (acute and late toxicity concerns) and radiation (time-dependent risk of developing secondary cancer) were considered last-resort alternatives. Because of the relatively aggressive growth kinetics, we suggested human IFN-α, referring to a retrospective Norwegian study (see details below) [13]. Although side effects of IFNs may be distressing, they typically attenuate within a few weeks after commencing treatment and dissipate within 2 weeks after termination.

It is speculated whether DTs are offshoots of an out-of-control repair process following tissue injury; hence, an immunological mechanism may well play a part in the pathophysiological process. This is supported by clinical effects of NSAIDs and IFN [12, 23, 24]. In the period 2008–2013, Poulsen *et al.* conducted a study at the Norwegian Radium Hospital using Multiferon®, a purified, multi-subtype IFN-α product containing α1, α2, α8, α10, and α21, in patients with locally advanced DTs. Daily doses of 3 million International Units (IU) × 6 days/week were injected subcutaneously. The outcomes in 18 treated patients were partial response in 5, stable disease in 11, and progressive disease in 1, and 1 was not evaluated [13]. Treatment time ranged from 8 to 38 months and resulted in symptom relief in the majority of patients.

Our patient received Multiferon® in similar daily doses for 4 months, resulting in disease stabilization. Side effects were flulike symptoms and malaise. With time, however, she developed a dry cough and dyspnea. Computed tomographic findings correlated with pneumonitis, a relatively rare but known severe side effect of IFN, which had to be stopped.

Suggested immunological activity in the lesion motivated further immunotherapy with local application to reduce the risk of systemic side effects. Weighing the risks of experimental treatment against chemotherapy or radiotherapy harmfulness, building on a favorable toxicity profile of LTX-315 in animal models and early human testing, the patient was thoroughly informed about and consented to participation in the phase I study of transdermal injection of the oncolytic peptide LTX-315 in solid tumors. Intratumoral LTX-315 resulted in reversal of tumor growth kinetics in parallel with gradual symptom relief and a slight decline in tumor diameter. Ongoing stable disease represents a successful long-term outcome.

Safety aspects in 61 patients receiving LTX-315 at doses of 2–7 mg/injection displayed 11 cases of grades 3–4 allergic reactions, the most serious (anaphylaxis) of which occurred after > 10 weeks of treatment, indicating development of specific antibodies over time with repeated drug exposure. This was taken into account in the amended protocol, restricting treatment time to a total of 3 weeks, compensated with multiple concurrent injections in one or more lesions. Commonly recorded adverse events were mild and transient hypotension, flushing, skin rash, and pruritus in approximately 50%. All allergy-like reactions typically subsided within approximately 30 minutes, with no late sequelae.

In summary, in cases of DTs interfering with daily function, action is warranted in order to control the disease. The therapeutic outcomes achieved with different drugs are unpredictable, although in general, the more toxic the therapy chosen from the armamentarium, the greater the anticipated effectiveness. In our patient, we opted for an experimental drug in a third-line setting (following NSAIDs and IFN-α). Occurrence of a severe allergic reaction led to termination of the study drug. Altogether, a 16-week period of LTX-315 therapy was followed by a gradual change in pain. Despite increased local tenderness during the treatment period, a long-term improvement of distress complemented by a slow reduction of tumor size suggested clinical benefit. A polyclonal expansion of PBMC was accompanied by an almost 17-fold increase in CD3^+^ T lymphocytes infiltrating the tumor tissue, transforming the immune gene expression from cold to hot and confirming activity of the drug. A slowly symptomatic recovery over several months in respect to improved sleep and physical functioning due to alleviated pain supports this conclusion. Further studies using local immunotherapy with LTX-315 in patients with DTs seem justified.
